# A case of Coffin–Siris syndrome with severe congenital heart disease and a novel *SMARCA4* variant

**DOI:** 10.1101/mcs.a003962

**Published:** 2019-06

**Authors:** Nikita R. Dsouza, Michael T. Zimmermann, Gabrielle C. Geddes

**Affiliations:** 1 Bioinformatics Research and Development Laboratory, Genomic Sciences and Precision Medicine Center, Medical College of Wisconsin, Milwaukee, Wisconsin 53226, USA;; 2 Clinical and Translational Sciences Institute, Medical College of Wisconsin, Milwaukee, Wisconsin 53226, USA;; 3 Department of Pediatrics, Medical College of Wisconsin, Milwaukee, Wisconsin 53226, USA;; 4 Herma Heart Institute, Children's Hospital of Wisconsin, Milwaukee, Wisconsin 53226, USA

**Keywords:** absent fifth toenail, complete atrioventricular canal defect, congenital sensorineural hearing impairment, obstructive sleep apnea

## Abstract

Coffin–Siris syndrome (CSS) is a developmental disability, caused by genomic variants in the gene *SMARCA4*, in addition to other known genes, but the full spectrum of *SMARCA4* variants that can cause CSS is unknown with 40% of cases not having molecular confirmation. In this report, we identify a patient with CSS, a severe cardiac phenotype, and a novel *SMARCA4* variant. There is no experimental structure of human SMARCA4, so we use molecular modeling techniques to generate a structural model of human SMARCA4. We then map known *SMARCA4* variants causative of CSS and our novel variant to the model. We use the resulting information to support the interpretation that the novel variant is causative of disease in our patient. Modeling demonstrates that the variant found in our patient is in a region of SMARCA4 associated with DNA binding, as are the other known pathogenic *SMARCA4* variants mapped. Because of this structural information, we discuss how these variants may be disease-causing through a dominant negative effect of disrupting DNA binding.

## INTRODUCTION

Coffin–Siris syndrome (CSS; #614609) is a syndromic form of cognitive and developmental disability most commonly associated with fifth finger hypoplasia and distinctive facial dysmorphisms including a wide mouth with thick, everted lips, thick eyebrows, and a broad nasal bridge consistent with facial coarseness (Schrier Vergano et al. 1993; Vergano and Deardorff 2014). Congenital anomalies of the brain, kidney, and heart have been described but are less consistent across patients. One-third of patients have congenital heart disease, typically including ventricular septal defects, atrial septal defects, Tetralogy of Fallot, and patent ductus arteriosus or patent foramen ovale (Schrier Vergano et al. 1993). More complex forms of congenital heart disease are not typically described (Nemani et al. 2014; Mannino et al. 2018).

CSS has been associated with de novo disruption of *ARID1A*, *ARID1B*, *SMARCA2*, *SMARCA4*, *SMARCB1*, *SMARCE1*, *SOX11*, and *PHF6* (Schrier Vergano et al. 1993), Pathogenic disruption of *SMARCA4* accounts for ∼7% of cases of CSS based on compilation of 172 cases reported in the literature (Schrier Vergano et al. 1993). SMARCA4 is an epigenetic regulator and chromatin remodeler of the SWI/SNF family of protein complexes. Disruption of SMARCA4 in patients with CSS are thought to be pathogenic through a dominant negative effect (Schrier Vergano et al. 1993). Patients with CSS due to disruption of *SMARCA4* seem to have an increased tendency to behavioral concerns, can have less coarseness to their facial features, and consistently have fifth digit and nail hypoplasia (Schrier Vergano et al. 1993).

As clinical genomic sequencing increases in use to diagnose rare disease such as CSS, we need novel approaches for variant interpretation. Mechanistic approaches are needed in order to be more confident in interpretation of variant information. Thus, we present a novel genomic variant likely causative of CSS and a mechanistic interpretation for SMARCA4 alteration.

## RESULTS

### Clinical Presentation

The patient was born at 39 wk gestation via vaginal delivery to a 33-yr-old G4 P3003 mother. APGARs were 8 and 9 at 1 and 5 min, respectively. Complex congenital heart disease characterized by a complete atrioventricular septal defect with a cleft mitral valve was diagnosed prenatally. A prenatal karyotype and chromosomal microarray were completed and normal. A two-vessel umbilical cord, ankyloglossia, and nonspecific dysmorphic features were noted at birth, and the child failed his newborn hearing screen. He underwent a genetic evaluation without additional testing completed at that time. After birth the cardiac anatomy was described as a slightly unbalanced complete atrioventricular septal defect with aortic root hypoplasia. There is no reported family history of congenital heart disease. The patient underwent complete cardiac repair at 5 months of age. The patient did not undergo routine or subspecialty follow-up outside of pediatric cardiology because of lack of familial resources and difficulty with coordination of complex care.

**Figure 1.**
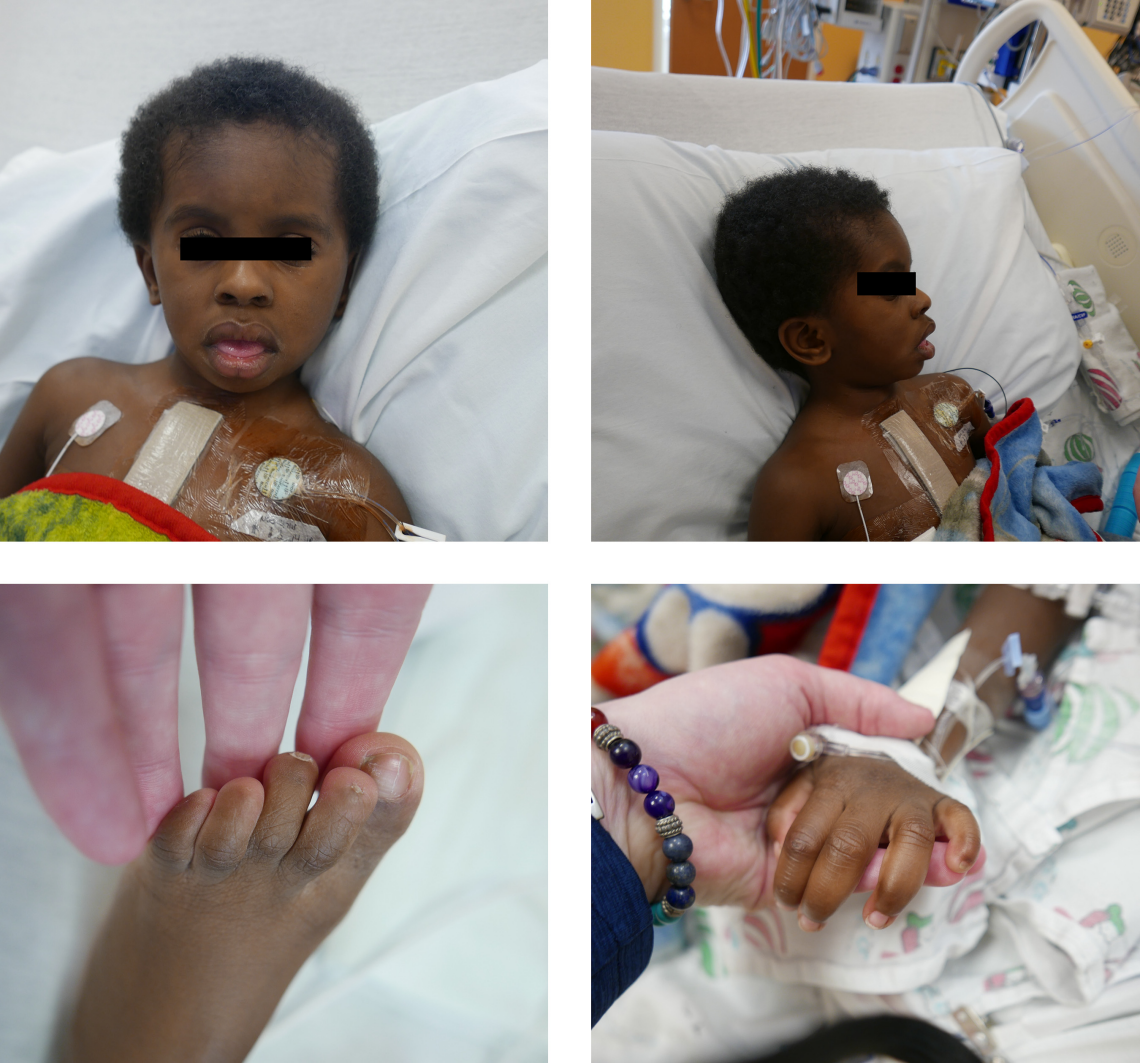
Patient at time of genetics evaluation following cardiac surgery. Patient has coarse facial features, spare bitemporal scalp hair, wide mouth with thick lips, and thick eyebrows, as well as low-set and posteriorly rotated ears. Fifth finger hypoplasia with nail hypoplasia is present. Nail hypoplasia and frank absence is present in the feet. Pictures published with specific permission from the patient's mother.

The patient presented to our hospital following family relocation from another state. First contact was at 34 mo of age with pediatric cardiology in which the patient was found to have progressive fibrotic subaortic stenosis as well as severe mitral regurgitation requiring further surgical intervention. At time of presentation to the genetics service the patient was 35 mo of age and had successfully undergone cardiac revision without residual hemodynamic dysfunction the day before. In addition to his cardiac anomalies he had a known history of left-sided hearing loss, obstructive sleep apnea, and global developmental delay without speech development. At time of initial genetics evaluation, a diagnosis of CSS was strongly suspected because of physical examination findings of coarse facial features, fifth digit distal hypoplasia, and nail aplasia/hypoplasia demonstrated in Figure 1. At this time, a clinical Coffin–Siris sequencing panel was completed.

### Genomic Analysis

The CSS panel included sequencing of *ADNP*, *ANKRD11*, *ARID1A*, *ARID1B*, *PHF6*, *SMARCA2*, *SMARCA4*, *SMARCB1*, *SMARCE1*, *SOX11*, and *TBC1D24*. A variant of uncertain significance in *SMARCA4* was reported (NM_001128849.1 c.2647G>A, p.Gly883Ser). The variant, demonstrated in the Table 1, was thought to be highly suspicious as it is in a known functional domain for SMARCA4, is at a highly conserved residue, was not found in allele frequency databases, and was predicted to be deleterious by multiple sequence-based software products including SIFT, PolyPhen-2, Align GVGD, and REVEL. Based on this information, we pursued a more detailed computational approach—molecular modeling—to assess the variant. This variant is in ClinVar under accession number SCV000891704.1. Father was not available for testing, and maternal testing was cost-prohibitive.

**Table 1.** Variant table

Gene	Chromosome	HGVS DNA reference	HGVS protein reference	Variant type	Predicted effect	dbSNP/dbVar ID	Genotype
*SMARCA4*	19p13.2	c.2647G > A	p.Gly883Ser	Substitution	Substitution	NA	Heterozygous

### Molecular Modeling

**Figure 2.**
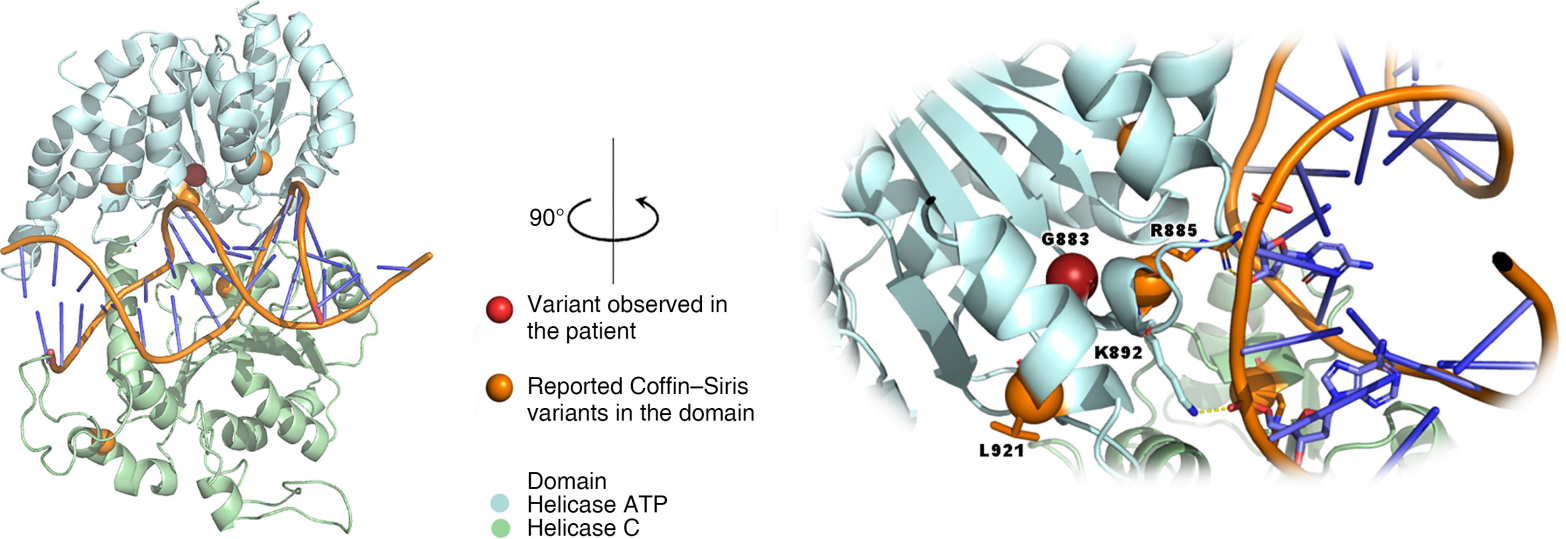
Variants that cause Coffin–Siris syndrome alter interactions between SMARCA4 and DNA. Shown here is the molecular model of the SMARCA4 bipartite helicase colored by the ATP and carboxy-terminal domains. Positions of known CSS variants are marked with orange spheres, and our novel case variant with a red sphere. The region around G883 where the sharp turn of the protein backbone is visible is enlarged here. A 90° rotated view shows the proximity of G883 to the likely position of bound DNA. The close relationship in 3D among CSS variants and the DNA binding surface is visually evident.

We used homology-based methods to build a model of DNA-bound human SMARCA4. Variants in *SMARCA4* known to cause CSS were mapped onto this model and compared to our patient's variant, providing further context shown in Figure 2. Variants in *SMARCA4* known to cause CSS were located within the same functional domain as our patient's variant. This domain binds to DNA. Reviewing the other *SMARCA4* variants known to cause CSS, we found that they and our patient's variant either alter the shape or electrostatic composition of the DNA binding site. In 3D, variants known to cause CSS are part of the DNA binding surface as demonstrated in Figure 2. Others are nearby to the DNA binding surface and likely function to help position the residues that make up the DNA binding surface. Variants either occurred at the DNA binding surface or were within the protein structure and likely forming the structural supports that position the DNA binding surface. Our patient's p.G883S variant specifically alters a sharp turn in the protein backbone which is accommodated by glycine, but not accommodated by serine as shown in Figure 2. The amino acid substitution likely alters the DNA binding site. This data strongly suggests this variant is causative of the phenotype observed in our patient.

We used a structure-based approach to compute the change in stability for our novel missense variant, p.G883S, and found it to be significantly destabilizing, ΔΔ*G*_fold_ = 5.4 kcal/mol. Because glycine is the only amino acid that readily enables the backbone flexibility needed for the native conformation, substitution to serine is highly destabilizing. All variants are either directly interacting with DNA or are destabilizing to the protein structure. This information supports that this variant significantly disrupts SMARCA4 structure and is causative of disease in our patient.

## DISCUSSION

This case presents a patient with Coffin–Siris syndrome with a complete atrioventricular septal defect due to a novel variant in *SMARCA4*. It is unclear to us at this time if the severity of the patient's cardiac phenotype is related to this specific variant or to other unidentified genetic factors. We propose based on our molecular modeling that the p.Gly883Ser *SMARCA4* variant is causative of CSS through disruption of the DNA binding site. This is a valuable insight for *SMARCA4* variant interpretation as variants in *SMARCA4* that alter DNA binding may be more likely to be causative of CSS. We believe the structural model assists in the interpretation of not only the observed genomic variants but also the potential mechanism of their dominant negative function. SMARCA4 is part of a protein complex. Thus, alteration of the DNA interaction may lead to a nonproductive SMARCA4 enzyme that may retain residual DNA binding capacity. Thus, it would bind to the same positions as the wild-type (wt) but would block enzymatic activity of even the wt protein complex.

A limitation of our case is we do not have functional cellular or biochemical evidence to support pathogenicity. We investigated existing literature for evidence of biochemical or cellular assays that could validate the predictions of our structure-based model; however, although many missense variants have been reported (Tsurusaki et al. 2014), unfortunately biochemical and cellular assessment lags behind genomic sequence-based detection. An additional limitation is the patient did not undergo exome sequencing to rule out the potential for a dual diagnosis explaining the severity of the cardiac phenotype.

In our future work we will explore additional genomic variants (e.g., from the CSS registry) and how our model may support variant interpretation. This would help us further understand how variants causative of CSS alter SMARCA4 structure and function and if this related to detectable phenotypic changes. We believe further mechanistic insight into how CSS occurs as a result of SMARCA4 disruption will allow for more complete *SMARCA4* variant analysis in addition to insight as to the effects genes that regulate gene expression have in humans.

## METHODS

### Molecular Modeling

There is no experimental structure of human SMARCA4. To build a SMARCA4 protein model, we first used sequence homology assessed by HMMER3 (Eddy 2009) to identify existing experimental structures that could be used as templates. The nucleosome-bound structure of SNF2 was identified as the best template (53% identical; 5X0X [Liu et al. 2017] in RCSB [Berman et al. 2000]). We built our model using this template and RaptorX (Källberg et al. 2014) homology modeling. We annotated our model with structural domains from UniProt (The UniProt Consortium 2017) and Superfamily (Kabisch et al. 2015). We used BioR (Kocher et al. 2014) to annotate genomic variants from ClinVar (Landrum et al. 2014) and the literature (Tsurusaki et al. 2012), and custom scripts to map them onto out protein structural model. We ran FoldX (Schymkowitz et al. 2005) to assess changes to the folding energy (ΔΔ*G*_fold_) of SMARCA4, specific to the observed variants.

Our model of SMARCA4 leveraged the nucleosome-bound SNF2 structure. Our model covers the helicase domain of SMARCA4; this domain is also ATP binding and has ATPase activity (Tsurusaki et al. 2012, 2014). Comparing our model to the SNF2 allowed us to map the protein region most likely to interact with DNA. We compared our SMARCA4 model to the SNF2 template and transferred a segment of the DNA bound to SNF2 to our SMARCA4 model. We used the proximity of amino acids and the orientation of side chains to make a simplified prediction for if each amino acid was likely to make interactions with the bound DNA or to act in supporting the DNA binding surface. To assist in the interpretation of variants observed in our clinical cases, we selected variants known to cause CSS. We used PyMOL version 2.0.7 for protein structure visualization, assessing the position of the variants in 3D and analyzing spatial patterns among structure-based scores. Our model is available as a protein data bank (PDB) file as Supplemental Information.

## ADDITIONAL INFORMATION

### Data Deposition and Access

The *SMARCA4* variant was submitted to ClinVar (http://www.ncbi.nlm.nih.gov/clinvar/) and can be found under accession number SCV000891704.1.

### Ethics Statement

Verbal and written consent were obtained from the patient's mother to participate in an Institutional Review Board reviewed project entitled “DNA Testing for Identifying Undiagnosed Genetic Disease.” Specific consent for publication, including publication of patient pictures, was obtained from the mother.

### Acknowledgments

The authors would like to thank the clinical team, the patient, and his family for supporting this project. We would also like to thank members of the Integrated Variant Analysis team, including Dr. Raul Urrutia and Dr. Donald Basel, for their support.

### Author Contributions

N.R.D. acquired and interpreted the data, drafted the primary manuscript, critically reviewed the manuscript, and approved the final manuscript. M.T.Z. and G.C.G. acquired and interpreted the data, critically reviewed the manuscript, and approved the final manuscript.

### Funding

This project had no direct funding.

### Competing Interest Statement

The authors have declared no competing interest.

### Referees

Samantha A. Schrier Vergano

Anonymous

## Supplementary Material

Supplemental Material
