# Supplementary material for: A case of Coffin–Siris syndrome with severe congenital heart disease and a novel SMARCA4 variant
Source: Cold Spring Harb Mol Case Stud. 2019 Jun;5(3):a003962. doi: 10.1101/mcs.a003962 (PMC6549553; doi:10.1101/mcs.a003962)
Supplement: Supplemental Material [file supp_5_3_a003962__index.html]

Supplemental Material 

# A case of Coffin–Siris syndrome with severe congenital heart disease and a novel *SMARCA4* variant

## Supplemental Material

- Supplemental\_Information.pdb
